# A genome-wide association study identifies a novel candidate locus at the *DLGAP1* gene with susceptibility to resistant hypertension in the Japanese population

**DOI:** 10.1038/s41598-021-98144-z

**Published:** 2021-09-30

**Authors:** Yasuo Takahashi, Keiko Yamazaki, Yoichiro Kamatani, Michiaki Kubo, Koichi Matsuda, Satoshi Asai

**Affiliations:** 1 Division of Genomic Epidemiology and Clinical Trials, Clinical Trials Research Center, Nihon University School of Medicine, 30-1 Oyaguchi-Kami Machi, Itabashi-ku, Tokyo, 173-8610 Japan; 2 Laboratory for Genotyping Development, RIKEN Center for Integrative Medical Sciences, Yokohama, Japan; 3 Laboratory of Complex Trait Genomics, Department of Computational Biology and Medical Sciences, Graduate School of Frontier Sciences, The University of Tokyo, Tokyo, Japan; 4 RIKEN Center for Integrative Medical Sciences, Yokohama, Japan; 5 Department of Computational Biology and Medical Sciences, Graduate School of Frontier Sciences, The University of Tokyo, Tokyo, Japan; 6 Division of Pharmacology, Department of Biomedical Sciences, Nihon University School of Medicine, 30-1 Oyaguchi-Kami Machi, Itabashi-ku, Tokyo, 173-8610 Japan

**Keywords:** Genetics research, Genetic association study, Hypertension

## Abstract

Numerous genetic variants associated with hypertension and blood pressure are known, but there is a paucity of evidence from genetic studies of resistant hypertension, especially in Asian populations. To identify novel genetic loci associated with resistant hypertension in the Japanese population, we conducted a genome-wide association study with 2705 resistant hypertension cases and 21,296 mild hypertension controls, all from BioBank Japan. We identified one novel susceptibility candidate locus, rs1442386 on chromosome 18p11.3 (*DLGAP1*), achieving genome-wide significance (odds ratio (95% CI) = 0.85 (0.81–0.90), *P* = 3.75 × 10^−8^) and 18 loci showing suggestive association, including rs62525059 of 8q24.3 (*CYP11B2*) and rs3774427 of 3p21.1 (*CACNA1D*). We further detected biological processes associated with resistant hypertension, including chemical synaptic transmission, regulation of transmembrane transport, neuron development and neurological system processes, highlighting the importance of the nervous system. This study provides insights into the etiology of resistant hypertension in the Japanese population.

## Introduction

Resistant hypertension is a medical disorder in which patients require at least four antihypertensive drugs of different classes for blood pressure control^1,2^, and is an increasingly important clinical problem. The exact prevalence of resistant hypertension is unknown. Data from clinical trials, however, suggest that it is not uncommon, affecting about 20 to 30% of patients with hypertension^3,4^. In the US National Health and Nutrition Examination Survey (NHANES), the prevalence of uncontrolled hypertension despite use of at least three antihypertensive drugs was reported to increase from 16% of patients treated for hypertension in 1998–2004 to 28% in 2005–2008^5^. Also, recent epidemiological research estimated that the prevalence of resistant hypertension is 8–12% of adult patients with hypertension^6^, and resistant hypertension occurs in 24% of treated hypertensive patients on hemodialysis^7^.

The etiology of resistant hypertension is unknown, but appears to be multifactorial^8^, with lifestyle-related, hormonal, and genetic factors having possible important roles. In addition to previously known risk factors, including older age and obesity, medical conditions such as impaired renal function and diabetes mellitus are also associated with resistant hypertension^4,9–11^. Elevation of circulating aldosterone level has been identified in the majority of patients with resistant hypertension^12–14^, drawing attention to the importance of aldosterone in the pathogenesis of resistant hypertension^15^. Also, genetic factors are believed to play a role in the disease etiology^16^. Whether the effects of genetic factors may differ between resistant hypertension and general hypertension is of interest. Although previous studies have identified numerous genetic variants associated with hypertension and blood pressure^17–23^, there is a paucity of evidence from genetic studies of resistant hypertension. The available genetic data regarding resistant hypertension are limited and primarily focused on candidate genes^24–29^. Additionally, pharmacogenomics research on resistant hypertension is in progress^16^. Recently, a few studies with a comprehensive genetic approach, genome-wide association studies (GWASs), have identified some significant loci for susceptibility to resistant hypertension in the US population^30–33^. However, there is still little evidence from genome-wide investigations of resistant hypertension, especially in the Asian population. Therefore, we performed a GWAS to identify novel genetic loci associated with resistant hypertension in the Japanese population.

## Results

### Genome-wide association study of resistant hypertension

**Figure 1:**
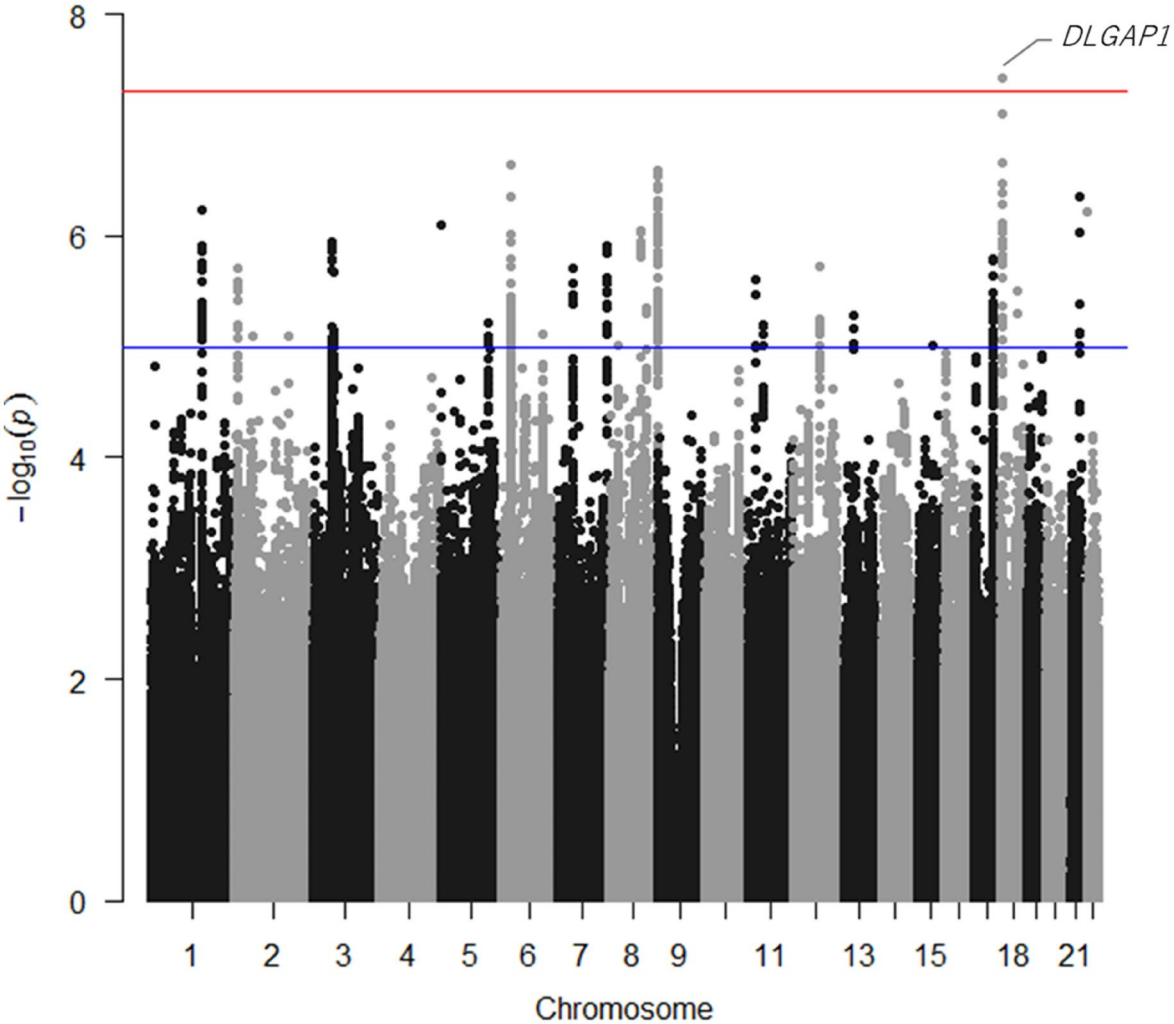
Manhattan plot for genome-wide association study (GWAS) of resistant hypertension in Japanese population. This plot is based on − log_10_(*P* value) from GWAS and imputation analysis against chromosome position. Blue line indicates suggestive association threshold, *P* < 1 × 10^−5^; red line indicates genome-wide significance threshold, *P* < 5 × 10^−8^.

**Figure 2:**
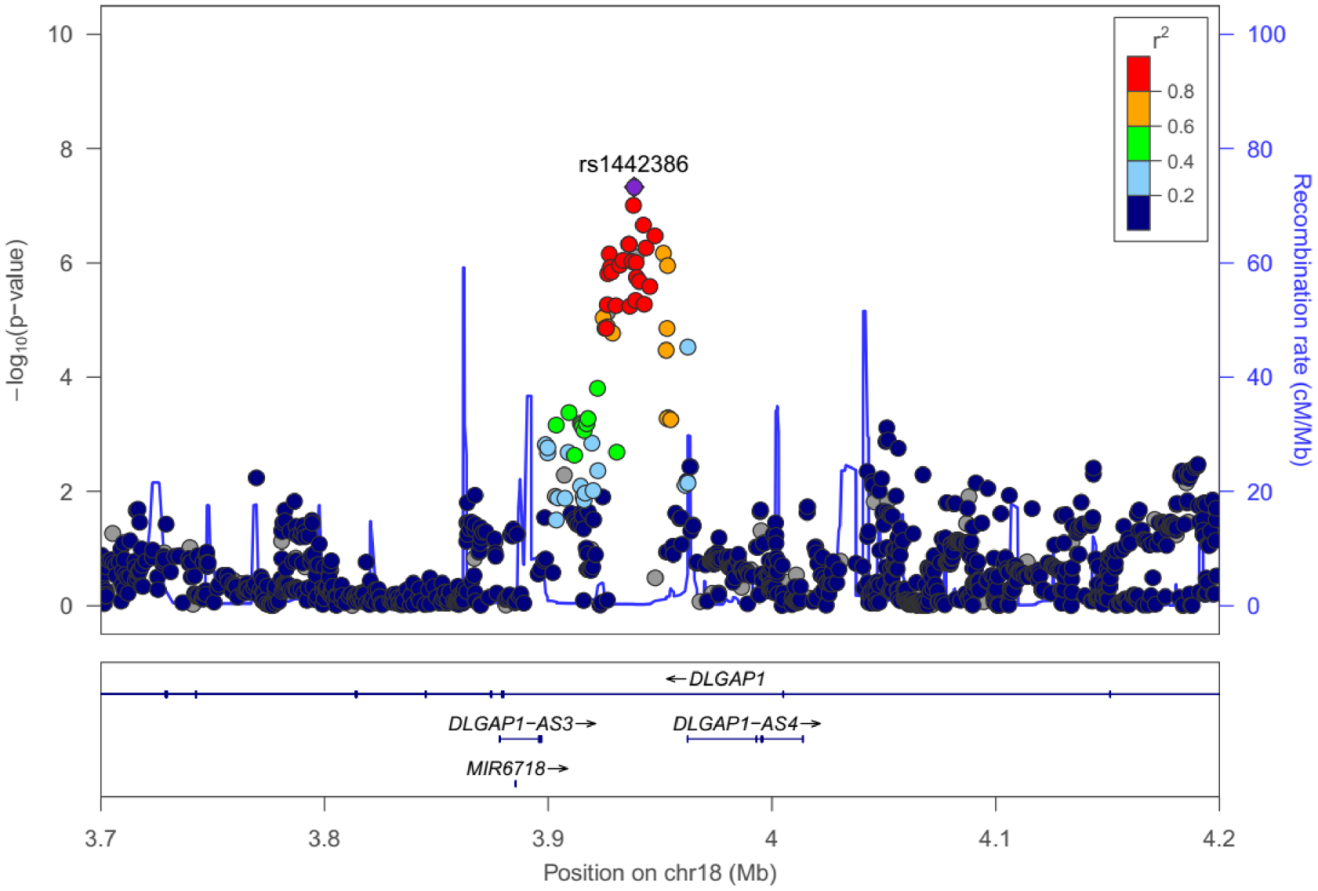
Regional association plots for locus significantly associated with resistant hypertension in Japanese population, on chromosome 18p11.3 (*DLGAP1*). The regional association plot was generated from the association data of a genome-wide association study (GWAS) with LocusZoom. Purplish blue lines represent local recombination rates. SNPs are colored according to their linkage disequilibrium (measured by *r*^*2*^) with the marker SNP. Diamonds (rs1442386) represent the most significantly associated SNP in each region in the GWAS. The SNP position is based on NCBI build 37.

To clarify the genetic architecture of resistant hypertension, we conducted a GWAS in a Japanese population consisting of 2705 resistant hypertension cases and 21,296 mild hypertension controls (Supplementary Fig. S1). We evaluated the possibility of population substructure for our sample population by comparison to HapMap samples using principal component analysis (PCA). Although all cases and controls were clustered in the Asian population, a very small portion of the samples was clustered in the Chinese population (Supplementary Fig. S2a and S2b). We then selected only samples from the major Japanese cluster for further analysis. After whole-genome imputation using the 1000 Genomes Project (1 KG) Phase 3 as a reference, we examined the association of 7,199,488 SNPs with minor allele frequency (MAF) of more than 1% and an estimated imputation accuracy of greater than 0.8. The quantile–quantile (Q-Q) plot shows the distribution of observed versus expected *P* values, while the corresponding genomic inflation factor (λ_GC_) of 1.048 suggests a low possibility of false-positive associations resulting from population stratification or cryptic relatedness (Supplementary Fig. S3). In addition, linkage disequilibrium (LD) score regression analysis, in which there was a low intercept of 1.0013, indicated low existence of substantial confounding bias, suggesting that the majority of the inflation was due to polygenic effects. The Manhattan plot, plotting − log_10_(*P* value) from the GWAS and imputation analysis against the chromosome position, is shown in Fig. 1. Our GWAS identified one genetic locus achieving genome-wide significance (*P* < 5 × 10^−8^) and 18 loci showing suggestive association (*P* < 1 × 10^−5^) with resistant hypertension in the Japanese population (Table 1). We examined each locus to determine whether it overlapped the 1 Mb-window of any variants that were previously reported to be significantly associated with blood pressure phenotypes, hypertension, or resistant hypertension. We detected one novel locus with significant association and three novel loci with suggestive association. The lead variant of the novel significant locus was rs1442386 (odds ratio (95% CI) = 0.85 (0.81–0.90), *P* = 3.75 × 10^−8^), which is located in the intron region of *DLG associated protein 1* (*DLGAP1*) on chromosome 18p11.3 (Fig. 2). The three novel suggestive loci were *PQLC3* (2p25), *LOC105369874* (12q14), and *MED4* (13q14) locus. The other 15 suggestive loci included variants previously reported to be associated with hypertension or blood pressure phenotypes. For example, variants of the *CYP11B2* locus have been frequently validated to be associated with hypertension in multiple populations^34,35^.

**Table 1:** Summary of association results of genome-wide association study in Japanese population.

SNP	Chr	Position	Nearest gene	RA	NRA	MAF		OR	L95	U95	*P*
		(bp)				Cases	Controls				
rs4072037	1	155,192,276	*MUC1*	C	T	0.196	0.171	0.85	0.79	0.91	5.30E−06
rs2075571	1	155,204,315	*THBS3*	C	T	0.211	0.184	0.84	0.79	0.90	1.24 E−06
rs3732103*	2	11,172,098	*PQLC3*	C	T	0.436	0.470	0.87	0.82	0.92	1.94 E−06
rs7625237	3	53,247,249	*TKT*	T	C	0.464	0.499	1.15	1.09	1.22	1.15 E−06
rs3774427	3	53,531,601	*CACNA1D*	C	G	0.141	0.120	1.21	1.11	1.31	6.13 E−06
rs253447	5	142,412,468	*SPRY4*	C	T	0.493	0.460	0.88	0.83	0.93	6.08 E−06
rs77163128	7	46,063,047	*IGFBP3*	T	G	0.286	0.257	1.16	1.09	1.23	1.93 E−06
rs79549409	7	151,157,081	*GBX1*	A	G	0.113	0.136	0.81	0.74	0.88	1.22 E−06
rs4247284	8	94,647,408	*ESRP1*	CA	G	0.276	0.245	0.85	0.80	0.91	8.85 E−07
rs62525059	8	142,901,545	*CYP11B2*	G	A	0.335	0.371	0.85	0.80	0.91	2.58 E−07
rs10833346	11	20,522,091	*PRMT3*	A	G	0.152	0.178	0.83	0.77	0.90	2.47 E−06
rs9787901	11	45,674,603	*CHST1*	G	A	0.386	0.418	0.87	0.83	0.93	6.25 E−06
rs200741614*	12	83,369,808	*LOC105369874*	G	T	0.096	0.078	1.25	1.14	1.38	1.89 E−06
rs11619475*	13	48,151,713	*MED4*	T	C	0.366	0.396	1.14	1.07	1.21	5.13 E−06
rs73324844	17	61,064,318	*BCAS3*	A	C	0.145	0.122	1.22	1.12	1.32	1.64 E−06
rs1442386*	18	3,938,439	*DLGAP1*	A	G	0.445	0.484	0.85	0.81	0.90	3.75 E−08
rs117652372	18	55,607,167	*TCF4*	G	A	0.100	0.083	1.22	1.11	1.35	3.14 E−06
rs2212606	21	38,673,016	*ERG*	A	C	0.298	0.333	0.85	0.80	0.91	4.48 E−07
rs78813487	22	17,007,603	*GAB4*	T	C	0.071	0.056	1.29	1.18	1.45	6.18 E−07

*SNP* single-nucleotide polymorphism (rsID of lead SNP); *Chr* chromosome, position, physical position of human genome version of GRCh38; *RA* risk allele, *NRA* non-risk allele, *OR* odds ratio, *L95* lower 95% confidence limit, *U95* upper 95% confidence limit, *MAF* minor allele frequency.

Odds ratios (OR) and confidence intervals (CI) were calculated using the non-risk allele as a reference. *:Indicates a novel locus for resistant hypertension. The nearest gene is shown as the locus label, but should not be interpreted as the best candidate. A list of all the genes in the 500-kb flanking region of the lead SNP is presented in Table S1.

### Functional annotation and expression quantitative trait loci (eQTL) analysis

We used HaploReg to perform functional analysis of a total of 19 lead variants showing association with resistant hypertension in the GWAS results. The functional annotations are summarized in Supplementary Table S2. rs4072037 at the *MUC1* locus was a synonymous variant, but the other 18 variants were located in non-coding regions (ten intronic and eight intergenic). Eight variants were located in gene expression regulatory motifs, such as enhancers, promoters, open chromatins and protein-binding sites in various tissue types. We found that several variants were identified as eQTLs of their nearest genes in various tissue types (Supplementary Table S3). Among them, two variants including rs4072037 and rs2075571 of 1q22 had associations (*P* < 0.05) with the expression levels of some genes, such as rs2075571 at the *THBS3* locus showing an association with *THBS3*, *GBA*, *RP11-263K19.6*, *GBAP1* and *MUC1* expression in various tissue types. Functional analysis of the rs1442386 variant which reached genome-wide significance showed one altered regulatory motif (GCM; glia cells missing) and a significant association with *DLGAP1* expression in whole blood at *P* = 0.0018. We also investigated the potential impacts of the lead variants associated with resistant hypertension on mRNA levels using the GTEx database of eQTL (Supplementary Table S4). We linked the GWAS association and the eQTL variant when the GWAS lead variant and the eQTL variant are in LD (r^2^ > 0.6 in East Asians of 1 KG Phase 3). We found that 13 suggestive signals can be explained by at least one eQTL variant, but seven variants including rs1442386 were not available in the GTEx database.

### Gene-based association analysis

We used VEGAS2 to obtain gene-based *P* values for phenotypic association from SNP-based *P* values, using the 1 KG EAS Phase 3 reference set. The genes for which the gene-based *P* values exceeded a Bonferroni-corrected threshold of *P* < 2.07 × 10^−6^ are given in Supplementary Table S5. Gene-based tests identified 21 genes associated with resistant hypertension, including *GBX1*, *AGAP3, ASB10, ABCF2* and *TMUB1* on chromosome 7q36, *ESRP1* and *LOC100288748* on 8q22, *CYP11B2*, *CYP11B, GML, LY6D, LYNX1_1, LYNX1_2* and *LOC100133669* on 8q24, *DLGAP1*, *DLGAP1-AS3*, *DLGAP1-AS4* and *MIR6718* on 18p11, *ERG* on 21q22, and *GAB4* and *CECR7* on 22q11. These genes were located not only at one locus with significant association, but also at five loci with suggestive association, in the current GWAS. For four lead variants, rs253447 of 5q31, rs77163128 of 7p12, rs200741614 of 12q14, and rs11619475 of 13q14, no genes were identified by VEGAS2, because these variants were intergenic and were located over more than 50 kb outside the neighboring genes, resulting in them being outside the subject for gene-based association analysis.

### Pathway-based association analysis

**Figure 3:**
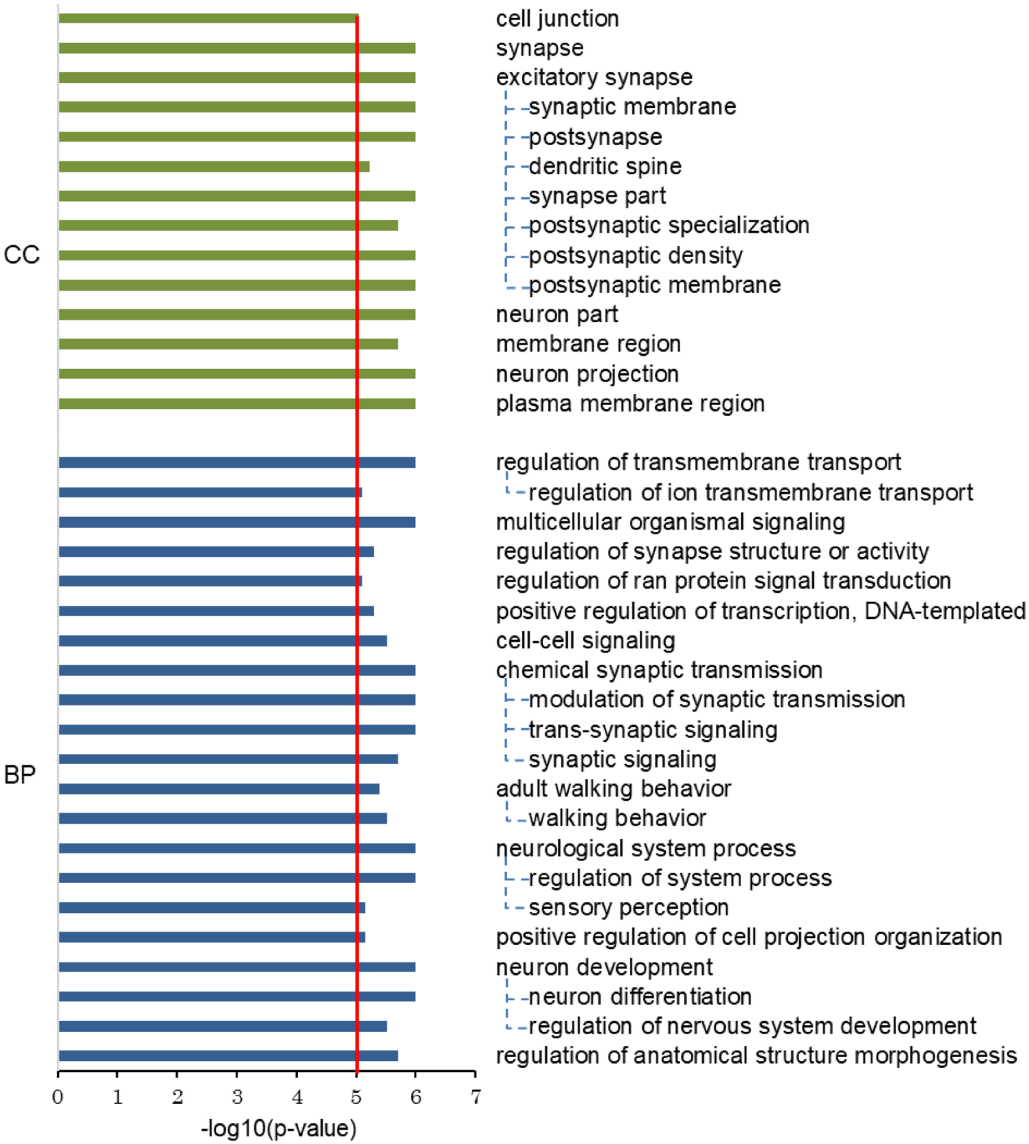
Results of significant pathways for resistant hypertension. Pathway analysis by VEGAS2Pathway revealed 35 significant pathways in cellular component (CC) (green) and biological process (BP) (blue). Red line indicates significance threshold of pathway-based *P* value (*P* < 1 × 10^−5^).

To further investigate the biological processes involved in resistant hypertension, we performed pathway-based association analysis using the VEGAS2Pathway approach. Figure 3 shows 35 Gene Ontology (GO) terms of biological process (BP) and cellular component (CC) that reached a genome-wide, pathway-based significant *P* value of less than 1 × 10^−5^ (Supplementary Table S6). Among them, we observed three prominent sets of GO terms that were highly associated with resistant hypertension. The most numerous set consisted of synapse (GO:0045202) and excitatory synapse (GO:0060076), especially involving postsynaptic compartments (CC term) for chemical synaptic transmission (GO:0007268) (BP term). Subsequently, a set of plasma membrane region (GO:0098590), postsynaptic membrane (GO:0045211), and membrane region (GO:0098589) for regulation of transmembrane transport (GO:0034762), and a set of neuron part (GO:0097458) and neuron projection (GO:0043005) for neuron development (GO:0048666) and neurological system process (GO:0050877) were also highly associated. These results suggest important pathways of the nervous system that may be involved in resistant hypertension.

### Comparison with previously reported variants

Previously reported variants showing an association (*P* in previous GWAS < 1 × 10^−5^) with resistant hypertension were evaluated in the current Japanese GWAS dataset (Supplementary Table S7). These 24 SNPs have been previously evaluated in five GWASs in multiple ethnic populations, including European, Hispanic, and African American subjects, but not in the Asian population^30–33,36^. These SNPs, however, did not show a significant association with resistant hypertension in the Japanese population. We further examined whether variants previously associated with blood pressure phenotypes or hypertension showed an association with resistant hypertension in the current GWAS dataset. A total of 2076 and 174 SNPs were selected as variants previously associated with blood pressure and hypertension from the NHGRI-EBI GWAS Catalog, respectively (*P* in previous GWAS < 5 × 10^−8^, listed in Supplementary Tables S8 and S9). Among these blood pressure-associated variants, eight at three loci showed suggestive associations with resistant hypertension (*P* < 1 × 10^−5^) (Supplementary Table S9). The most significant association was rs62525059 of 8q24 at the *CYP11B2* locus (*P* = 2.58 × 10^−7^). The next suggestive associated variants were rs4072037 of 1q22 near *MUC1/GBAP1* (*P* = 5.30 × 10^−6^) and rs3774427 of 3p21 near *CACNA1D* (*P* = 6.51 × 10^−6^). Also, two loci, including rs62525059 (*CYP11B2*) and rs3774427 (*CACNA1D*), showed a suggestive association with resistant hypertension in variants previously associated with hypertension (Supplementary Table S9), the same as those previously associated with blood pressure. These results suggest the possibility that *CYP11B2* (the aldosterone synthase gene) and *CACNA1D* (a member of the voltage-gated calcium channel gene family) may be involved in the development not only of hypertension, but also of resistant hypertension.

Replication analysis in the same population is of importance for genetic studies. Because we could not prepare replication cohorts in a Japanese population for resistant hypertension, we conducted a validation study using previous GWAS results. However, GWAS summary statistics of a Japanese population for resistant hypertension or hypertension were not publicly available. Therefore, we utilized summary statistics of multi-ethnic populations for hypertension. We conducted validation analysis at 19 loci comprising 1 significant and 18 suggestive associations in our GWAS. Among 19 variants, 10 were shown to have the same effect direction (Supplementary Table S10). Meta-analysis using a fixed-effect model indicated that one variant (rs3774427 near *CACNA1D*) passed the genome-wide significance level (*P* value < 5 × 10^−8^) after combining the GWAS and the validation study, with no significant heterogeneity (*P* value > 0.05) between the two GWAS datasets. However, this validation study was not successful in reproducing the *DLGAP1* results observed in our GWAS for resistant hypertension. This reproducibility failure in multi-ethnic populations suggests that further efforts to conduct a replication analysis in a Japanese population will be required to confirm the associations in this study. In addition, the current GWAS data that were used to assess the association with resistant hypertension did not successfully detect a large number of previous GWAS findings. Most of the previously reported variants associated with blood pressure were established from studies of quantitative traits of blood pressure phenotypes. In addition, previous GWASs of hypertension frequently adopted non-hypertensive subjects or general populations as the control. On the contrary, the present study evaluated a binary outcome using mild hypertensive controls, which may have led to a reduction in statistical power. These differences in our GWAS data may have resulted in the discrepancy in genetic correlations from previous findings in quantitative outcomes or studies using non-hypertensive controls.

## Discussion

To investigate novel susceptibility loci for resistant hypertension, we performed a GWAS in the Japanese population consisting of 2705 resistant hypertension cases and 21,296 controls. We identified a novel locus at chromosome 18p11.31 (*DLGAP1*) associated with resistant hypertension that reached genome-wide significance. Also, we identified 18 loci with suggestive association, 14 of which were previously reported to be associated with hypertension or blood pressure (e.g. the *CYP11B2* or *CACNA1D* locus).

The lead SNP (rs1442386) of the most significant association locus achieving genome-wide significance in this study was in the intron region of the *DLGAP1* gene, which is exclusively expressed in brain and encodes disks large-associated protein 1^37^. This variant is putatively GCM motif-altering, and is significantly associated with *DLGAP1* expression in whole blood cells (Supplementary Table S2). The DLGAP1 protein, which localizes at postsynaptic density and interacts with postsynaptic density 95 (PSD95) protein, is involved in signaling at neuronal postsynaptic densities and maintaining normal brain function and development^37^. Although the findings of pathway analyses remain putative, our analyses showed that chemical synaptic transmission and regulation of transmembrane transport, including synaptic and trans-synaptic signaling pathways in the synapse and postsynaptic compartments, were significantly associated with resistant hypertension. Additionally, we observed significant associations of neuron development and neurological system process pathways with resistant hypertension. Our GWAS findings, combined with pathway analyses, provide an insight that the DLGAP1 protein at the postsynaptic membrane in the central nervous system may contribute to driving resistant hypertension, and highlight the importance of the nervous system in the etiology of resistant hypertension. Genetic variations of *DLGAP1* are known to be associated with several psychiatric disorders, such as obsessive–compulsive disorder, schizophrenia, and major depressive disorder^38–40^. A worldwide epidemiological study has shown that resistant hypertension is associated with mental stress and anxiety^41^. These findings suggest that psychological stress may play a possible role in the pathophysiology of resistant hypertension. Further studies are needed to examine the associations between resistant hypertension and anxiety disorders and the possible roles of each in the development of the other.

The lead SNP (rs62525059) of the next suggestive association locus was located 9 kb downstream of *CYP11B2* (the aldosterone synthase gene), which is the rate limiting step of aldosterone synthesis in humans^42^. This variant was reported to be associated with blood pressure and hypertension in East Asian populations, including Japanese^43,44^. In addition, our study revealed that some variants at the CYP11B2 locus, which were reported to be associated with both hypertension and blood pressure, showed a suggestive association with resistant hypertension in this GWAS. Furthermore, gene-based association analysis in this study revealed that the *CYP11B2* gene was significantly associated with resistant hypertension, supporting the GWAS results. Previous genetic studies of resistant hypertension revealed the association of variants related to the aldosterone and aldosterone pathways, including the beta and gamma subunits of the epithelial sodium channel (*ENaC*)^26,45^, angiotensinogen (*ATG*)^46^, and *CYP4A11*^47^. Regarding clinical features of resistant hypertension, elevation of circulating aldosterone level has been identified in the majority of patients with resistant hypertension^12–14^, suggesting that excessive aldosterone is involved in the pathophysiology of resistant hypertension^12^. Our findings in the Japanese population, in combination with previous genetic and clinical findings, suggest that the aldosterone synthase gene (*CYP11B2*) may be a potential causal gene for resistant hypertension, and support the important role of aldosterone and its pathways in the pathophysiology of resistant hypertension, the same as for hypertension.

The *CACNA1D* gene, encoding the alpha-1D subunit of the calcium channel, showed suggestive evidence of an association with resistant hypertension (Table 1). Calcium channel proteins are involved in a variety of calcium-dependent processes, including hormone or neurotransmitter release, and gene expression^48^. *CACNA1D* is a member of a family of voltage-gated calcium channel genes, several of which are a target of calcium channel blockers. The lead SNP (rs3774427) at the *CACNA1D* locus was reported to be associated with both hypertension and blood pressure in the GWAS Catalog, and showed a suggestive association with resistant hypertension in this GWAS (Supplementary S8, S9 and S10). In addition, our pathway analyses showed that regulation of transmembrane transport including regulation of ion transmembrane transport were strongly associated with resistant hypertension. Our findings suggest that a pathway for regulation of transmembrane transport, via the calcium channel, may play an important role not only in blood pressure regulation, but also in the development of resistant hypertension.

Our GWAS data did not successfully replicate *PTPRD* findings in previous studies on resistant hypertension^30–33^ (Supplementary Table S6). This discrepancy may derive in part from differences in race, selection of cases and controls, sample size of each study, and prescription rate for each class of antihypertensive drugs. The prescription rates for β-blockers in the non-resistant hypertension group were approximately 50 percent in previous GWASs^30,33^, but was approximately 5 percent in this study (Supplementary Table S11). As the *PTPRD* gene is associated with blood pressure response to atenolol^32^, this difference in the prescription rate of β-blockers may be the reason that previous GWASs discovered an association with resistant hypertension in the *PTPRD* region, and that we could not find a susceptibility locus near the *PTPRD* gene. On the other hand, our GWAS discovered a suggestive association with resistant hypertension in the *CACNA1D* and *CYP11B2* regions using a Japanese population, with high prescription rates for calcium channel blockers and angiotensin receptor blockers. These data suggest that the genetic feature of resistant hypertension can be complex because of a combination of multiple susceptibility genes associated not only with excessive blood pressure, but also with drug resistance. Our study, including pathway analysis, can explain only one aspect of the phenotype of resistant hypertension from a drug resistance perspective. GWAS stratified by classes of antihypertensive agents may be useful to validate this assumption. We will examine this theme in our next study.

Our study has some potential limitations. First, there is a possibility that diagnostic misclassification, which may impact on estimation of genetic correlations of GWAS^49^, may have occurred in our study. Because data on the diagnosis of hypertension were not available in this study, we defined patients with resistant hypertension based on antihypertensive drug prescription data. Also, data on blood pressure were limited to the first entry, resulting in incomplete evaluation of blood pressure control after the initiation of antihypertensive medications. Therefore, we adopted a more stringent diagnosis, defined as patients who had received four or more classes of drugs whatever their blood pressure control. However, this definition has the concern of potential systematic exclusion of uncontrolled patients who had received three classes of antihypertensive drugs. Second, regarding the control group, we identified patients with mild hypertension, defined as patients who had received one antihypertensive drug for at least 1 year. This definition may cause misclassification bias in that control samples may have included patients with moderate or severe hypertension, even if a small number of patients, leading to underestimation of genetic correlations. Also, we might have missed some positive results. These concerns call for further studies, such as replication studies using larger Japanese samples with diagnosis by physicians, to confirm the validity of our findings. Third, data on detailed clinical information were not available in this study, and we could not account for them. In addition, the model used in this study was not adjusted for BMI because of a large number of missing data. The possibility that this may have impacted on our results cannot be excluded. Fourth, the ethnicity of all our study subjects was Japanese, and cases were ethnically matched with the controls, limiting the ability to generalize the results. Further studies are needed to confirm the association of the locus and resistant hypertension in other cohorts of different races, because the relevance of our findings to other ethnic groups remains to be demonstrated.

We identified a novel candidate locus at the *DLGAP1* gene with susceptibility to resistant hypertension with genome-wide significant levels, and 18 loci, including *CYP11B2* and *CACNA1D*, as having suggestive association with resistant hypertension in the Japanese population. Pathway analysis revealed that chemical synaptic transmission, regulation of transmembrane transport, neuron development and neurological system processes are significantly associated with resistant hypertension. The DLGAP1 protein is known to be involved in signaling at neuronal postsynaptic densities and in maintaining normal brain function. Our novel findings suggest a possible role of *DLGAP1*, which may contribute to susceptibility to resistant hypertension, possibly via the central nervous system, leading to a new target for drug discovery in the future. In addition, our data suggest that *CYP11B2* (the aldosterone synthase gene) and *CACNA1D* (a member of the voltage-gated calcium channel gene family), which are known to be associated with hypertension, may be involved in the development not only of hypertension, but also of resistant hypertension, and underline the importance of these loci in resistant hypertension. Further replication studies are essential to confirm the findings.

## Materials and methods

### Data source

The genotyping data and clinical information in this GWAS were obtained from BioBank Japan. The BioBank Japan project, which began in 2003, is a collaborative network of 66 hospitals in all areas of Japan that has collected genomic DNA, serum and clinical information from approximately 270,000 patients diagnosed with any of 51 diseases^50,51^. All subjects received a detailed explanation, and all signed a written informed consent form. The study protocol conformed to the ethical guidelines of the Declaration of Helsinki and was approved by the Ethics Committees of all participating institutions, including the Institute of Medical Science, the University of Tokyo, the Center for Integrative Medical Sciences, RIKEN, and Nihon University School of Medicine.

### Study populations and phenotype definitions

A flowchart outlining the identification of study subjects is shown in Supplementary Fig. S1. First, we examined prescription data, which were collected annually from 2003 to fiscal 2012 in the BioBank Japan Project. We identified 78,463 Japanese patients with hypertension, defined as patients who had received at least one prescription of any antihypertensive drug between 2003 and 2012. A list of antihypertensive drugs included in this study is presented in Supplementary Table S12. Direct alpha antagonists such as phentolamine and phenoxybenzamine were excluded from this drug list because they are mainly used for the treatment of pheochromocytoma and hypertensive emergencies^31^. Then we selected study subjects, aged over 40 years at enrollment, with resistant hypertension as cases and mild hypertension as controls, fulfilling the following criteria:

1)The resistant hypertension group was defined as patients who had received four or more classes of antihypertensive drugs for at least 1 year.

2)The control group was defined as patients who had received one antihypertensive drug for at least 1 year, and who had never received two or more antihypertensive drugs.

To exclude patients who discontinued a medication shortly after initiation, we selected patients who had received any antihypertensive drug for 1 year or more by evaluating prescription records for at least 2 years. Consequently, we identified a total of 25,450 patients with hypertension (3978 cases and 21,472 controls) who fulfilled the above criteria, and their prescription rates for each class of antihypertensive drugs are listed in Supplementary Table S11. After quality-control and PCA described in detail below, Japanese subjects comprising 2705 cases and 21,296 controls were used to conduct a GWAS. The demographics of the study subjects at enrollment are summarized in Table 2. We incorporated age and sex as covariates for GWAS as described below. However, we did not use body mass index (BMI) as a covariate because of a large number of missing data of BMI near the prescription date in the study population.

**Table 2:** Baseline demographics of study population.

	Cases	Controls	*P* value
Number of samples	2705	21,296	
Women (%)	35.8%	42.1%	< 0.0001
Age (years)	65.8 ± 10.5	66.4 ± 10.9	0.0053
BMI (kg/m^2^)	25.4 ± 4.07	23.6 ± 3.5	0.0001
**Blood pressure (mmHg)**			
Systolic	139.9 ± 18.5	131.5 ± 15.6	0.0001
Diastolic	79.1 ± 12.4	76.5 ± 10.5	0.0001

*BMI* body mass index; *SD* standard deviation.

Data at enrollment are summarized. Data are mean ± SD unless otherwise stated. Comparisons of patient demographics between cohorts were performed using Welch’s *t* test for continuous variables and chi-squared test for categorical data.

### Genotyping and imputation

Genotyping was performed using the Illumina HumanOmniExpressExome BeadChip or a combination of the Illumina HumanOmniExpress and HumanExome BeadChips. We aligned the probe sequence in the manifest files of the genotyping array to the GRCh37.3 reference using BLAST to convert genotypes into forward strands. For sample quality control, we excluded samples with (i) sample call rate < 0.98, (ii) closely related samples identified using identity by state (IBS) using PLINK^52^, or (iii) outliers from the East Asian Cluster identified by PCA using the genotyped samples and the three major reference populations (Africans, Europeans, and East Asians) in the International HapMap Project by smartpca^53^. Then, we applied quality control for genetic variants and excluded SNPs with (i) SNP call rate < 0.99 in both cases and controls, or (ii) Hardy–Weinberg equilibrium *P* ≤ 1 × 10^−6^ and MAF < 1%.

We pre-phased the genotypes with SHAPEIT^54^ and imputed dosages with IMPUTE2^55^ using 1 KG Phase 3 as a reference^56^, which was supplied by IMPUTE2 site. For subsequent analysis, we used genotypes with an imputation quality of info ≥ 0.8 and MAF ≥ 1%. A Q-Q plot was constructed using observed *P* values against expected *P* values and an inflation factor value (λ-value) that was calculated to assess potential population stratification of the study subjects^57^. LD score regression was conducted using LDSC^58^ version 1.0.1 and the East Asian LD scores of the 1 KG Project, to estimate the extent of inflation from confounding bias.

### Analysis of GWAS data

For general statistical analysis, we used R statistical environment version 3.4.3 or PLINK1.07. GWAS was used to perform association analysis using imputed allele dosages by snptest^59^. The association model was adjusted for age and sex. We set the threshold for genome-wide significance at the level of *P* < 5 × 10^−8^ and the threshold for suggestive significance at *P* ≤ 1 × 10^−5^. A Manhattan plot was generated using R software to visualize the results. Regional association plots were generated using LocusZoom^60^. The online tool HaploReg v4.1 was used to explore the genes nearest to the index SNPs, and genes containing a missense mutation in high LD (r^2^ > 0.8) with the GWAS SNPs^61^. The effects of GWAS SNPs on expression in eQTL studies of different tissues were extracted from the query results of HaploReg or GTEx^62^ portal v8.

We defined an associated locus as a genomic region within ± 500 kb from the lead SNP. We excluded SNPs in the major histocompatibility complex region (chromosome 6: 26–34 Mb). We defined a locus as novel when it did not overlap the 1 Mb-window of any variants that were previously reported to be significantly associated with blood pressure phenotypes, hypertension, or resistant hypertension (P in previous GWAS < 5 × 10^−8^).

### Evaluation of previously reported variants

We searched for previously reported variants showing an association (*P* in previous GWAS < 5 × 10^−8^) with hypertension or blood pressure phenotypes (including blood pressure, systolic blood pressure, diastolic blood pressure, and mean arterial pressure), or an association (*P* in previous GWAS < 1 × 10^−5^) with resistant hypertension, in the GWAS Catalog (https://www.ebi.ac.uk/gwas/, accessed July 2020) and the literature^30,31,33,36^. We evaluated our GWAS statistics of these previously reported variants.

### Validation of findings in this GWAS by previous GWAS

We searched GWAS summary statistics for hypertension or resistant hypertension that are publicly available. We obtained summary statistics for hypertension (PMID. 31217584)^63^ in multi-ethnic populations including Hispanic/Latino, African American, Asian, Native Hawaiian, Native American ancestry subjects from the GWAS Catalog. We extracted statistics of this previous GWAS at the lead variants with significant or suggestive association with resistant hypertension in our Japanese GWAS, and compared our statistics and those reported in the previous GWAS. We considered a previous GWAS signal as replicated when the signal in the previous GWAS had the same effect direction in our GWAS. Meta-analysis was conducted using a fixed effect model with inverse-variance weighting, and tested heterogeneity in effect size estimates using Cochran’s Q test.

### Gene and pathway-based analysis using VEGAS2 software

We performed gene-based association testing using VEGAS2 (version 2) software^64^. VEGAS2 is an extension of the VErsatile Gene-based Association Study approach which uses 1000 genomes as reference data to estimate linkage disequilibrium between variants within a gene. Based on SNP association *P* values of GWAS data, the software calculated empirical gene-based *P* values by a simulation procedure. We performed analysis using 1 KG Phase 3 East Asian populations. Gene boundaries were set to ± 50 kb of each gene. Up to 10^6^ simulations were performed per gene. A total of 24,098 genes were tested, and genes with *P* < 2.07 × 10^−6^ (Bonferroni correction for multiple testing, i.e., 0.05/24,098) were considered to be significantly associated with resistant hypertension. Subsequently, we performed pathway analysis using the VEGAS2Pathway approach^65^. VEGAS2Pathway performs pathway-based association testing and calculates empirical *P*-values of association for each pathway, while accounting for LD between variants within a gene and between neighboring genes, gene size, and pathway size by using resampling of gene-based test statistics. The Biosystems gene-pathway annotation file was obtained from the VEGAS2 official site (https://vegas2.qimrberghofer.edu.au/biosystems20160324.vegas2pathSYM). The significance threshold of the empirical *P*-value in the pathway analysis was set at 1 × 10^−5^ while taking into account the multiple testing of correlated pathways (0.05/5000 independent tests)^64^.

## Supplementary Information

Supplementary Information 1.

Supplementary Information 2.

## Data Availability

Individual genotyping data and clinical information that support the findings of this study are publicly available at the National Bioscience Database Center with accession code hum0014 (http://humandbs.biosciencedbc.jp/).
