# Supplementary material for: A genome-wide association study identifies a novel candidate locus at the DLGAP1 gene with susceptibility to resistant hypertension in the Japanese population
Source: Sci Rep. 2021 Sep 30;11:19497. doi: 10.1038/s41598-021-98144-z (PMC8484335; doi:10.1038/s41598-021-98144-z)
Supplement: Supplementary file 1 — Supplementary Information 1. [file 41598_2021_98144_MOESM1_ESM.docx]

**Supplementary Information**

A genome-wide association study identifies a novel candidate locus at the *DLGAP1* gene with susceptibility to resistant hypertension in the Japanese population

Yasuo Takahashi^1^

Keiko Yamazaki^1, 2^

Yoichiro Kamatani^3^

Michiaki Kubo^4^

Koichi Matsuda^5^

Satoshi Asai^1, 6^

^1^Division of Genomic Epidemiology and Clinical Trials, Clinical Trials Research Center, Nihon University School of Medicine, Tokyo, Japan.

^2^Laboratory for Genotyping Development, RIKEN Center for Integrative Medical Sciences, Yokohama, Japan.

^3^Laboratory of Complex Trait Genomics, Department of Computational Biology and Medical Sciences, Graduate School of Frontier Sciences, The University of Tokyo, Tokyo, Japan.

^4^RIKEN Center for Integrative Medical Sciences, Yokohama, Japan.

^5^Department of Computational Biology and Medical Sciences, Graduate School of Frontier Sciences, The University of Tokyo, Tokyo, Japan.

^6^Division of Pharmacology, Department of Biomedical Sciences, Nihon University School of Medicine, Tokyo, Japan.

Correspondence should be addressed to:

Yasuo Takahashi, M.D., Ph.D.

E-mail: yasuo.takahashi@nihon-u.ac.jp

Division of Genomic Epidemiology and Clinical Trials, Clinical Trials Research Center, Nihon University School of Medicine, Tokyo, Japan

or

Satoshi Asai, M.D., Ph.D.

E-mail: asai.satoshi@nihon-u.ac.jp

Division of Pharmacology, Department of Biomedical Sciences, Nihon University School of Medicine, Tokyo, Japan.

Supplementary Figures

78,463 patients with hypertension were identified for this study, fulfilling the following criteria (#1).

Allocation to patients, aged over 40 years at enrollment, with resistant hypertension as cases and mild hypertension as controls, fulfilling the following criteria.

1. Resistant hypertension group was defined as patients who had received four or more classes of antihypertensive drugs for at least one year (#2).
2. Control group was defined as patients who had received one antihypertensive drug for at least one year, and who had never received two or more antihypertensive drugs.

53,017 not meeting the criteria excluded.

25,446 patients, aged over 40 years, included in this study.

Resistant hypertension (N= 3,974), control (N= 21,472).

1,445 excluded fulfilling the following criteria:

(1) sample call rate < 0.98, (2) closely related samples identified using identity by state (IBS), or (3) outliers from the Japanese Cluster using principal component analysis (PCA) for genotype (#3).

24,001 Japanese patients, aged over 40 years, included in this GWAS.

Resistant hypertension (N= 2,705), control (N= 21,296).

**Supplementary Figure S1. Identification of study population.**

(#1) Inclusion criteria: Genotyping data and clinical information were obtained from BioBank Japan. We examined prescription data, which were collected annually from 2003 to fiscal 2012 in the BioBank Japan Project. Subjects with hypertension, defined as patients who had received at least one prescription of any antihypertensive drug between 2003 and fiscal 2012, were identified for the study.

(#2) Patients were assigned to case and control groups based on antihypertensive drug prescription data. To exclude patients who discontinued a medication shortly after initiation, we selected patients who had been prescribed any antihypertensive drug for one year or more by evaluating prescription records for at least two years.

(#3) BioBank Japan provides genotyping data of Japanese (mainly) subjects, but does not exclude subjects of East-Asian descent. We therefore excluded patients who were not of Japanese origin through PCA for genotype.


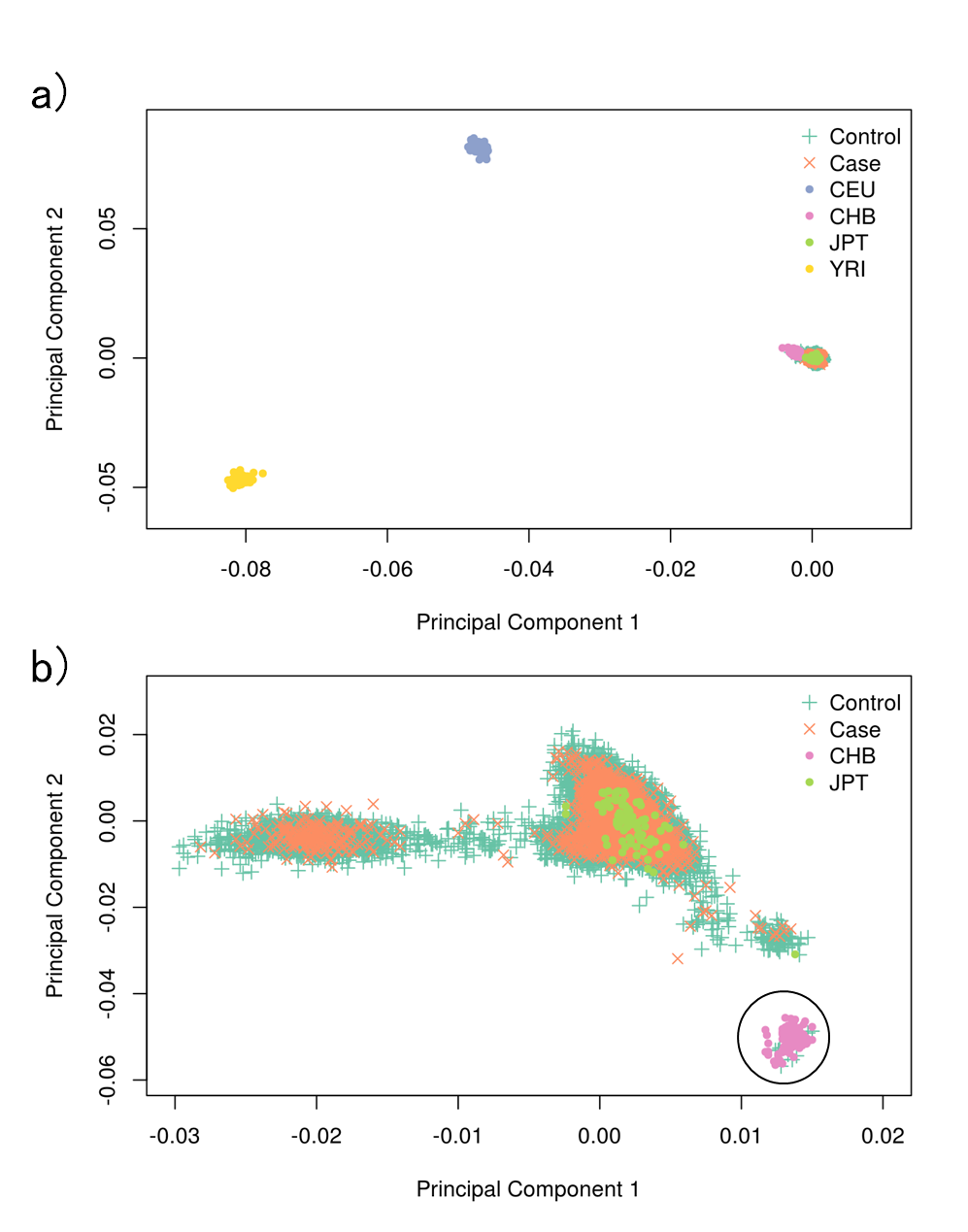


**Supplementary Figure S2. Results of principal components analysis of population in genome-wide association study.**

(a) The relatedness among cases and controls for GWAS along with European (CEU), African (YRI), and East-Asian (JPT and CHB) data from the HapMap Project was analyzed. (b) Relatedness, along with East-Asian (JPT and CHB) data from the HapMap Project, was analyzed. Samples clustered in the Chinese population (within the black circle) were omitted from further analysis.


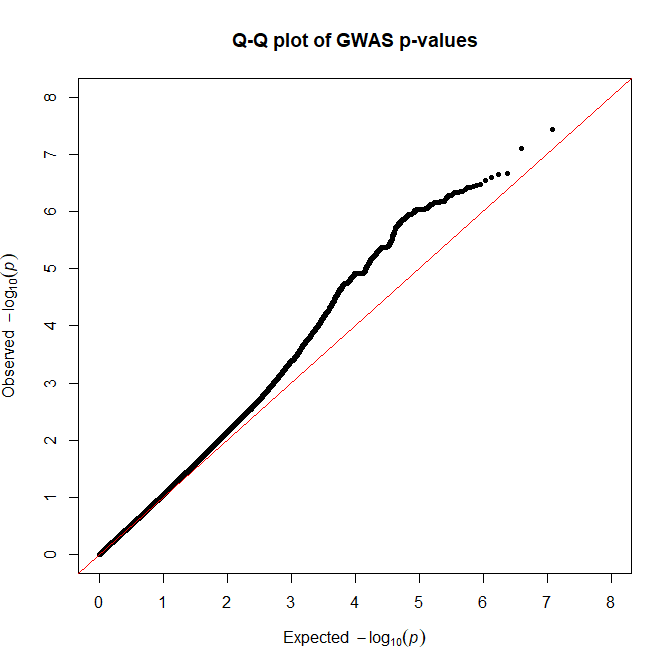


**Supplementary Figure S3. Quantile-quantile (Q-Q) plot of *P* values for genome-wide association study of resistant hypertension in Japanese population.**

The genomic inflation factor (lambda) value is 1.048.
